# Research Advances in Identifying Sulfate Contamination Sources of Water Environment by Using Stable Isotopes

**DOI:** 10.3390/ijerph16111914

**Published:** 2019-05-30

**Authors:** Huiwei Wang, Qianqian Zhang

**Affiliations:** Hebei and China Geological Survey Key Laboratory of Groundwater Remediation, Institute of Hydrogeology and Environmental Geology, Chinese Academy of Geological Sciences, Shijiazhuang 050061, China; whuiwei@mail.cgs.gov.cn

**Keywords:** water environment, sulfate, stable isotope technique, source apportionment

## Abstract

As the main anion of groundwater, the content of sulfate affects the drinking water safety and ecological security directly. In recent years, with the acceleration of industrialization and urbanization development, the problem of sulfate pollution in water environments is becoming more and more serious. It is critical to effectively identify the sulfate sources of water environment to ensure human health and the benign evolution of water environment. Due to its “fingerprints” feature, the sulfur and oxygen isotopes of SO_4_^2−^ have been widely used to identify sources of sulfate contamination in water environment. However, research advances in tracing sulfate contamination sources of water environment by using stable isotopes are rarely reported. This paper reviewed the research advances of sulfate isotope technology domestically and abroad, which was used to trace the sources of sulfate pollution in water environment, compared different pre-treatment methods for analyzing the δ^34^S and δ^18^O of sulfate, and compiled the ranges of typical values of δ^34^S and δ^18^O from different potential sources of sulfate contamination. In this review, the limitation of the technique in traceability of sulfate pollution was also discussed, and the future traceability techniques of sulfate pollution were prospected.

## 1. Introduction

Sulfate, as a common anion in the water environment, is widely distributed in various natural environments and plays an important role in biogeochemical cycles. However, as a constant component in the water environment, its pollution problem is often neglected. In recent years, with the development of industrialization and urbanization, sulfate contamination in the water environment has become increasingly prominent, receiving more and more attention from managers and researchers [1].

The increasing concentration of sulfate in the water environment not only threatens human health and ecological balance [2], but may also affect carbonate weathering, erosion processes, and global carbon cycle evolution [3,4]. Previous studies have shown that, when the human body take in excessive sulfate, it will cause several diseases, e.g., diarrhea, dehydration, and gastrointestinal disorders, etc. [5]. Sulfate in the water environment may be transformed into the toxic substances under certain conditions, resulting in the loss of essential metal elements in aquatic plants and changes in the original eco-hydrological function. Soucek et al. have shown that high concentrations of sulfate will cause the death of freshwater invertebrates [6]. The highly sulfate concentration of water environment not only influences human life, but also places some constraints on industrial water and irrigation water. Therefore, the WHO and China in Sanitary Standard for Drinking Water Quality limit the sulfate concentration less than 250 mg/L [7].

Identifying the sources of sulfate contamination accurately is the premise of controlling the sulfate pollution in the water environment. Dissolved sulfate in water environment is primarily derived from both natural and anthropogenic sources [8]. Natural sources include dissolution of sulfate minerals (e.g., gypsum), oxidation of sulfide minerals (e.g., pyrite), precipitation and volcanic activity, etc. Anthropogenic sources contain sewage infiltration, fertilizers, synthetic detergents, industrial wastewater and mining drainage, and so on. In addition, groundwater over-exploitation will accelerate the sulfate pollution [5,9,10]. The diversity of sulfate sources and its effects on the ecological environment are attracting more and more researchers’ attention to distinguish the sulfate sources and determine the mechanisms of sulfur and oxygen isotopic variations of SO_4_^2−^ in different water and the control factors. Therefore, study on the sources of sulfate in water environment is of great significance to water environment safety.

The traditional method to trace the sulfate sources is combining the geological background of the study area with the hydrochemical characteristics, which is simple, but the accuracy is poor. With the advancement of the science and technology, scholars have found that the SO_4_^2−^ from different sources has particular δ^34^S and δ^18^O values and thus it has been widely used to identify sulfate sources and the processes of sulfur biogeochemical cycles [11,12,13,14].

Based on previous researches, the author reviews the current research progress of identifying the sources of sulfate pollution in water environment by using the δ^34^S and δ^18^O isotope technology. The main contents are as follows: (1) The fractionation mechanism of sulfur and oxygen isotope of sulfate is introduced, (2) the ranges of typical values of δ^34^S and δ^18^O from different potential sources of sulfate are complied, (3) the research advances of sulfate isotope technology in domestic and abroad, which was used to trace the sources of sulfate contamination in water environment are summarized, and (4) the future traceability techniques of sulfate in water environment are prospected.

## 2. Stable Sulfur and Oxygen Isotopes and the Kinetic Isotope Fractionation

There are four natural stable isotopes of sulfur: ^32^S (95.02%), ^33^S (0.75%), ^34^S (4.21%), and ^36^S (0.02%). The sulfur isotope composition is usually characterized by the relative abundance of ^32^S and ^34^S, using the troilite (FeS) (CDT) from the Canyon Diablo in United States as a standard. Oxygen possesses three stable isotopes: ^16^O (99.759%), ^17^O (0.037%), and ^18^O (0.204%). The traditional reference is Vienna Standard Mean Ocean Water (VSMOW). The sulfur isotope fractionation mechanism is mainly divided into equilibrium isotope fractionation and kinetic isotope fractionation.

The stable isotope ratio is usually expressed as *δ*, which is the stable isotope ratio relative to the standard, the expression is as follows:

*δ*_sample_ (‰) = [(R_sample_ − R_standard_)/R_standard_] × 10^3^ (1)

where R is the isotope ratio, which is the ^34^S/^32^S or ^18^O/^16^O of the sample or standard.

Due to the different ability of different substances to enrich S and O, isotope fractionation often exists when the state of matter changes. The fractional degree is often expressed by the isotopic fractionation coefficient:

α_A-B_ = R_A_/R_B_ (2)

where R_A_, R_B_ represent A, B material isotope ratio, respectively. In addition, the enrichment factor is defined as:

ε = α_A-B_ − 1 = (R_A_/R_B_ − 1) × 10^3^ (3)

The kinetic isotope fractionation mainly occurs in the unidirectional chemical and biochemical processes. Among them, the unidirectional chemical reaction is common in the precipitation, dissolution and adsorption/desorption processes of sulfate minerals and the oxidation of reduced sulfur (S^0^, HS^−^, H_2_S, FeS_2_). However, the sulfur isotope fractionation of these processes is relatively small. On the contrary, the microbial reduction of sulfate during biochemical processes will result in larger sulfur isotope fractionation.

## 3. Pretreatment Technology

The pretreatment technology of sulfur and oxygen isotope samples mainly include graphite reduction method, fluorination method, high temperature pyrolysis method, chemical precipitation method, triacid method, and flame heating method, etc.

### 3.1. Graphite-Reduction Method

The graphite-reduction method, proposed firstly by Rafter in 1967, is the traditional oxygen determination method in sulfate and is widely used all over the world. The BaSO_4_ is reduced with graphite at 1100 °C to produce CO_2_ and CO. The CO is determined directly or converted to CO_2_, and then analyzed by mass spectrometry (MAT-253EM, Key Laboratory of Isotope Geology of Ministry of Land and Resources, Institute of Mineral Resources, CAGS, Beijing, China). The method is simple and the results are more accurate, and the test precision is ±0.2‰ [15].

### 3.2. Fluorination Method

The method comprises the following steps: the sulfate is reacted with a strong oxidizing agent such as fluorine gas or fluorine halide (e.g., BrF_5_) under high temperature to generate O_2_; the generated O_2_ is converted into CO_2_ at 700 °C in a graphite furnace; then, the oxygen isotope composition is measured, and the analytical uncertainty is about ±0.17‰ [16]. This method can also directly measure the oxygen isotope composition in O_2_, which can simultaneously determine the δ^18^O and δ^17^O value [17,18]. Among them, when using F_2_ as oxidant, because of its low purity, a small amount of oxygen, inconvenience in operation and poor safety, this method has less application. While BrF_5_ has strong oxidation properties, it is liquid at normal temperature, and its thermal stability is well, so it is used as the oxidant in determining the oxygen isotope values widely. However, compared with the graphite reduction method, the analysis process is relatively complicated, and the oxygen yield is low, and the measured δ^18^O value still needs to be corrected.

### 3.3. High-Temperature Pyrolysis Method

The samples are pyrolytically decomposed at 1400 °C in the presence of nickelized graphite to produce CO, and the volatile product is separated by a gas chromatography column to directly measure the δ^18^O value in CO. This method is simple and convenient, and it can be used to determine the δ^18^O values in inorganic and organic samples on-line. However, for some carbonate samples, the CO production rate is low, the measurement results are inaccurate, sulfates of 50–100 μg O can be analyzed for δ^18^O, and the standard deviation is better than ±0.5‰ [19].

### 3.4. Chemical Precipitation Method

The collected water samples (approximately 1.5 L) are filtered through 0.45 μm cellulose-acetate membrane filters, and then acidified to pH ≤ 2 with HCl. The SO_4_^2−^ of samples is precipitated as BaSO_4_ by adding excess 10% BaCl_2_. The obtained barite was rinsed with deionized water to remove Cl^−^, filtered and dried for 2 h at 850 °C. The δ^34^S and δ^18^O in BaSO_4_ was determined by the element analyzer (Carlo Erba 1108, School of Environmental Studies, China University of Geosciences, Wuhan, China) and isotope mass spectrometer (Delta C Finningan Mat, School of Environmental Studies, China University of Geosciences, Wuhan, China) and the analytical precision for δ^34^S is better than ±0.2‰ [20]. In order to improve the purity of barite, the prepared BaSO_4_ is dissolved and reprecipitated by DTPA reagent (diethylenetriaminepentaacetic acid, [(HO_2_CCH_2_)_2_NCH_2_CH_2_]_2_-NCH_2_CO_2_H). The purified BaSO_4_ is purified and analyzed by isotope ratio mass spectrometry (Finnigan MAT 253, Louisiana State University, Baton Rouge, LA, USA), and the test precision of δ^18^O is ±0.5‰ [21,22]. The dissolution and reprecipitation (DDARP) method can remove the nitrates and other impurities in BaSO_4_, which makes the determination of oxygen isotope more accurate.

### 3.5. Triacid Method

For this method, the mixed solution of HCl, HI and H_3_PO_2_ are used to react with the sulfate minerals to obtain Ag_2_S, which is oxidized to SO_2_ directly, and the δ^34^S value is determined by the mass spectrometry (MAT-230C, Institute of Mineral Resources, Chinese Academy of Geological Sciences, Beijing, China). The chemical process is complicated and inconvenient to operate [23]. This method is mainly suitable for sulfate minerals.

### 3.6. The Flame Heating Method

In the flame heating method, sulfate mineral samples are semi-melted by Na_2_CO_3_-ZnO, and the sulfate samples are transformed into BaSO_4_. Then BaSO_4_ and SiO_2_ are heated by the flame in a vacuum to generate SO_3_, which is following reduced by copper to form SO_2_ to determine the δ^34^S values. This method generates a large amount of harmful gas during the combustion process which is dangerous, and consumes a large amount of quartz tubes. In order to eliminate the drawbacks of the method, BaSO_4_ is mixed with SiO_2_ and V_2_O_5_ in a tube furnace under high-temperature heating at a vacuum environment to obtain SO_2_ for δ^34^S measurements [24]. The test accuracy (MAT 251 EM, Institute of Mineral Resources, Chinese Academy of Geological Sciences, Beijing, China) is better than ±0.2‰ [25].

At present, the pretreatment method commonly used for the determination of sulfate isotope is chemical precipitation method which is combined with elemental analyzer and stable isotope mass spectrometer, the sulfur and oxygen isotope values of sulfate can be determined. The graphite reduction method, the fluorination method, and the pyrolysis method can determine the oxygen isotope values of the sulfate samples, and the triacid method and the flame heating method are used to determine the sulfur isotope value in the sulfate samples.

## 4. Sulfur and Oxygen Isotope Values of Sulfate from Different Sources

Sulfate in groundwater mainly originates from the atmosphere, pedosphere, lithosphere and anthropogenic sources of pollution. The SO_4_^2−^ from the atmosphere, biosphere and anthropogenically sources can enter the aquifer through the infiltration and recharge processes, while the lithosphere sulfur can enter the aquifer as a result of the water-rock interaction. Each source has its own sulphur and oxygen isotope characteristics.

### 4.1. δ^34^S Values of Sulfate Sources

The pollution status of sulfate in various water bodies is relatively common. Moreover, due to the smaller isotope fractionation (except bacterial reduction) during the biogeochemical cycle of sulfur, sulfur isotopes of sulfate are widely used to identify the pollution sources of sulfate in water environment. Previous studies showed that δ^34^S values originating from atmospheric deposition are between −3–12‰, the typical δ^34^S values for fertilizers are situated between −7–21‰, from detergent are −3.2–25.8‰ [26], from evaporites are −14–35‰, and from pyrite ranges from −15‰ to 4‰ [27].

**Figure 1:**
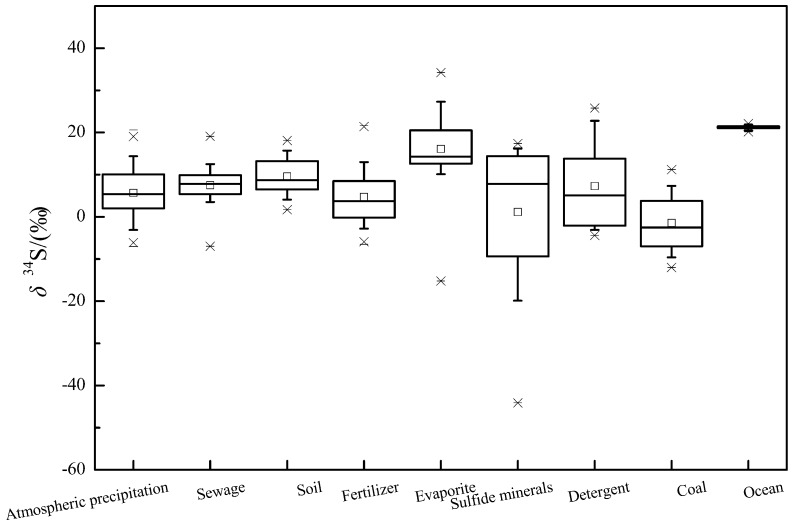
Box plots of *δ*^34^S values of SO_4_^2−^ from various sources. (1) The small box in the box-plots represents the average value, the straight line illustrate the median value, the upper and lower borders of the box diagram represent the 25% and 75% of the *δ*^34^S values, and the whisker indicate the 10% and 90% of the *δ*^34^S values. (2) Data sources-[8,9,12,26,28,29,30,31,32,33,34,35,36,37,38,39,40,41,42,43,44,45,46,47,48,49,50,51,52,53,54,55,56,57,58,59,60]; Sample size (*n*): Atmospheric precipitation = 264, sewage = 38, soil = 49, fertilizer = 115, evaporite = 264, sulfide minerals = 118, detergent = 41, coal = 100, and ocean = 28. The different colors from the left to the right refer to the atmospheric precipitation, sewage, soil, fertilizer, evaporite, sulfide minerals, detergent, coal, ocean, respectively.

In recent years, researches on the use of sulfur isotope to trace the source of sulfate pollution have emerged. In this paper, nearly 50 literatures about sulfur isotopes of sulfate were collected, and the ranges of δ^34^S-SO_4_^2−^ values from different sources were summarized (Figure 1).

In this paper, the main pollution sources of sulfate are roughly divided into atmospheric deposition, soil, fertilizer, evaporite, sulfide mineral, detergent and coal. Among them, the isotopic composition of sulfur in atmospheric deposition is mainly affected by natural and anthropogenic activities (such as the burning of fossil fuels). In China, studies have shown that atmospheric precipitation exhibited significant spatial distribution characteristics, which the precipitation in the North part of the Yangtze River is mainly enriched in the heavier sulfur isotopes and the values are mostly positive, while the South part is the opposite [35]. As shown in Figure 1, the typical δ^34^S values range of the atmospheric precipitation (10% and 90% of the Box-whisker Plot) is between −3.2–14.4‰, with a mean value of 5.7‰.

The δ^34^S values of sewage and agricultural fertilizes are significantly affected by the geological conditions, local human activities and the different sources of fertilizer raw materials [8,12]. The typical δ^34^S values for sewage and fertilizes are situated between 2–12.5‰ and −3.2–13‰, with an average of 7.4‰ and 4.7‰, respectively (Figure 1).

The sulfur isotope composition in soil is mainly affected by the type of sulfur-containing substances in the soil, biological processes(mineralization of organic sulfur and dissimilatory sulfate reduction) and abiotic effects(migration and transformation of sulfides) [61]. During the dissimilatory microbial sulfate reduction, the sulfate-reducing bacteria are more inclined to utilize the lighter isotopes which resulting in the enrichment of δ^34^S in the residual sulfate, while the reduced products are enriched in δ^32^S [24,62,63,64]. According to Figure 1, the typical range of δ^34^S in soil is between 4.1–13.6‰, with a mean value of 9.6‰.

When the stratum contains evaporites (such as gypsum), the water body usually has a high SO_4_^2−^ concentration and δ^34^S value during the water-rock interaction process, and the δ^34^S value in the gypsum is positive [65,66]. The isotope compositions based on the geological age [67]. For sulfide minerals, SO_4_^2−^ formed by oxidation generally has a negative δ^34^S value, which is affected by the oxygen isotope composition of oxygen sources (H_2_O and O_2_) and its contribution during oxidation [65,68]. According to Figure 1, the typical δ^34^S values for evaporites and sulfide minerals ranges from 9.5‰ to 28.3‰ and −25‰ to 16.2‰, with mean values of 16.1‰ and 1.15‰, respectively.

The sulfur isotope composition in detergents is primarily attributed to different sources of the raw materials that provide S. Generally powder detergents have a relatively higher δ^34^S value [12]. It is known from Figure 1, the typical δ^34^S values for detergents ranges from −3.2‰ to 22.8‰, with a mean value of 7.3‰. The value of sulfur isotope in modern oceans is relatively narrow, with a typical range of 20.5–21.7‰ (the average is 21.24‰).

Due to the different genesis of coal in different regions, the sulfur isotope composition is quite different. In China, coal in the Northern regions has a relatively positive δ^34^S value and lower sulfur content, while the South is opposite [60]. As shown in Figure 1, the typical values of δ^34^S in coal range from −9.9‰ to 7.3‰, with a mean value of −1.5‰.

### 4.2. δ^18^O Values of Sulfate Sources

Since when the main sources of sulfate in the water environment are traced by the single δ^34^S isotope, there will be overlaps in the range of δ^34^S values from different sulfate sources, such as atmospheric precipitation and sulfide oxidation sources, sulfate bacterial reduction processes and gypsum dissolution, etc. [28,69]. Therefore, utilizing the single δ^34^S value has constrains on identifying the main sources of sulfate in water environment. In order to trace the sulfate sources accurately, researchers gradually have begun to use the sulfate oxygen isotope to trace the sources together.

The oxygen isotopic composition of sulfate is mainly affected by the weathered zone, the oxidation reaction pathway, sulfate bacteria reduction and the isotopic composition of local water [24]. At present, the researches on the oxygen isotopes of sulfate pollution sources are relatively few, which mainly focused on atmospheric deposition, soil, chemical fertilizers, and detergents. Previous studies showed that the δ^18^O values of sulfate in chemical fertilizers are 7.7–16.5‰, δ^18^O values in detergents are 11.2–20.6‰, and δ^18^O values in atmospheric deposition are 5–17‰ [26]. The δ^18^O values in sulfide oxidation are from −5–4‰, while in gypsum it ranges from 14.5‰ to 32.5‰ [69].

**Figure 2:**
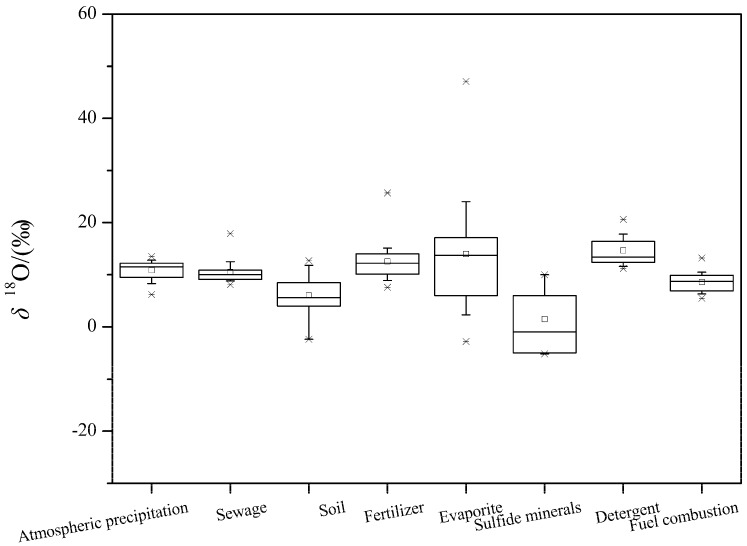
Box plots of *δ*^34^O values of SO_4_^2−^ from various sources. (1) The small box in the box-plots represents the average value, the straight line illustrate the median value, the upper and lower borders of the box diagram represent the 25% and 75% values, and the whisker indicate the 10% and 90% of the *δ*^34^O values. (2) Data sources-[10,26,28,29,32,39,40,43,46,47,48,49,65,66,68,70,71,72,73,74,75,76,77]; Sample size (*n*): atmospheric precipitation = 66, sewage = 31, soil = 10, fertilizer = 42, evaporate = 44, sulfide minerals = 6, detergent = 12, and fuel combustion = 11.

In this paper, approximately 30 literatures about oxygen isotopes of sulfate were summarized, including the atmospheric precipitation, sewage discharge, soil, chemical fertilizer, evaporites, sulfide minerals, detergents, and fuel combustion sources. The results are shown in Figure 2.

The δ^18^O values in atmospheric deposition are mainly affected by natural and anthropogenic activities (such as fuel combustion), and have a relatively positive value of δ^18^O [28]. As shown in Figure 2, the typical δ^18^O value of atmospheric deposition is situated between 7.7–12.8‰ (average 11‰); the typical δ^18^O value of fuel combustion ranges from 5.5‰ to 10.5‰, with an average value of 8.6‰. The typical δ^18^O values for soil S, evaporites and sulfide minerals are situated between −2.4–11.8‰, 1.1–24‰, and −5.17–6‰, with an average of 6.01‰, 13.98‰, and 1.47‰, respectively (Figure 2).

The typical δ^18^O values of sulfate from sewage, fertilizers and detergent (powder and liquid) fall into the ranges of 8.2–12.5‰, 8.8–15.1‰, and 11.2–17.8‰, respectively, while the average value is 10.5‰, 12.5‰, and 14.7‰, respectively.

## 5. Research Advances in the Application of Sulfur and Oxygen Stable Isotopes in the Identification of Sulfate Sources in Water Environments

The stable isotope techniques for the identification of sulfate sources at home and abroad have been studied for more than 40 years. It has gone through the processes from studying the mechanisms of sulfate isotope fractionation to identify sulfate sources qualitatively and quantitatively. In the early research stage, scholars mainly relied on the traditional hydrochemistry techniques to analyze and discuss the sources and pollution mechanism of sulfate. With the development of science and technology, stable isotope technology developed gradually, and sulfate sulfur isotope technology was widely used to trace the sources of sulfate in water environment [9,52,53,78]. Mizota et al. [45] found that the δ^34^S values of soils and fertilizers were generally higher in areas of the Southern Hemisphere than the Northern Hemisphere which were due to the differences in the relative contribution of S from marine aerosols and anthropogenic activities. Otero et al. [8] used sulfate sulfur isotopes to study the effects of potassium mining on groundwater salinization in Llobregat Basin. The results showed that the main sources of sulfate pollution were tailings water and fertilizers. Yang et al. [79] investigated the main sources of sulfate in the Ordos Cretaceous Groundwater Basin and the results showed that the sulfate in groundwater was mainly derived from gypsum and mirabilite in the stratum, followed by sulfide in the stratum and a small amount from organic sulfur. Hosono et al. [46] used complex isotope (H, O, N, S, Sr) techniques to identify the main sources of sulfate in different water bodies from different regions. The main sources of sulfate in karst groundwater in Guiyang were studied by using sulfur and chlorine isotopes and the results showed that the main sources of sulfate in groundwater were the dissolution of gypsum, the influence of coal-bearing stratum, the dry and wet atmospheric deposition, and the organic sulfur oxidation in soil [9,78].

Since there may be overlaps in the δ^34^S values of sulfate from different sources, therefore, researchers began to pay more attention to simultaneous determination of sulfate sulfur and oxygen isotopes in the sulfate sources identification [28,69]. Dowuona et al. [71] traced the sulfate sources in Southern Saskatchewan (Canada) by using sulfate sulphur and oxygen isotopes, and the results showed that the sulfate in this region was derived from sulfides. Yang et al. [70] studied the main sulfate sources in the the Ordos Cretaceous Groundwater Basin by using the S and O isotopes. It was identified that the sulfate in shallow groundwater was mainly derived from atmospheric precipitation, sulfides oxidation, and sulfate minerals dissolution, while in the deep groundwater, was the dissolution of sulfate minerals. Li et al. [76] used a dual isotopic approach to trace sulfate sources in Changjiang Estuary, China, and the results indicated that atmospheric deposition, dissolution of evaporate and oxidation of sulfide minerals were the main sources of water sulfate in this area. The δ^34^S and δ^18^O values of the groundwater sulfate in the Caldas da Rainha Spas indicated that the sulfate were the result of water-rock interaction with evaporitic rocks (e.g., gypsum and anhydrite) [80]. Al-Charideh et al. [81] traced the main sources of sulfate in carbonate aquifer system in Aleppo basin (North Syria) based on sulfate sulfur and oxygen isotopes. Xiao et al. suggested that the high SO_4_^2^^−^ concentrations in the geothermal water resulted mainly from the dissolution of gypsum according to the δ^34^S and δ^18^O values [82].

With the deepening of research, scholars have generalized models of the sulfate sources contribution rate in different water environments based on the principle of mass balance. Miao et al. [74] used stable isotope techniques, combined with geochemical and hydrogeological data, to explore that groundwater sulfate sources at a mining site (The Monument Valley site in Arizona, AZ, USA) and calculate the sources contribution rates by using the quantitative models. Samborska et al. [73] found that nearly 50% of the sulfate was derived from the weathering of sulfide minerals, the second largest source is atmospheric precipitation (accounting for 30%), and the rest comes from the dissolution and evaporation of sulfate minerals by utilizing the models. The sources apportionment contribution model of sulfate sulphur and oxygen isotopes can be generalized to [83]:

(4) $$\delta^{34}S\;=\;\overset{n}{\underset{i=1}{\sum }}f_{i}\;\times \;\delta^{34}S_{i}$$

(5) $$\delta^{18}O=\overset{n}{\underset{i=1}{\sum }}f_{i}\;\times \;\delta^{18}O_{i}$$

(6) $$1=\overset{n}{\underset{i=1}{\sum }}f_{i}$$

Among them, *i* represents different pollution sources, δ^34^S_i_ and δ^18^O_i_ represent the δ^34^S and δ^18^O values of sulfate in the pollution source *i*, and *f_i_* represents the contribution rate of different pollution sources.

## 6. Research Deficiency and Prospect 

As a constant component in water environment, the problem of excessive sulfate concentration is often neglected. With the continuous development of the economy and society, the problem of sulfate pollution in the water environment has become increasingly prominent, which has been widely concerned by scholars. Comprehensive analysis of traceability of sulfate contamination at home and abroad shows that although the traceability technology for sulfate pollution in groundwater has been developed, the accuracy of traceability still needs to be further improved. There are mainly several deficiencies in the following aspects: First, previous studies on sulfate pollution in water environment are mainly based on the hydrochemistry theory, or only the application of the sulfate δ^34^S isotope with poor accuracy of tracing results, and mainly concentrated in the qualitative researches. In the later stage, researches on the sulfate contamination traceability gradually developed into dual and multiple isotopes traceability, while the comprehensive tracing method and quantitative research are not mature. Second, previous researches on sulfate pollution mainly focus on surface water and rainwater, and there are relatively few studies on groundwater sulfate pollution, especially in areas where human activities are relatively intensive. Third, the determination of the end element values of potential sulfate pollution sources is mostly based on the data in the literature, and it lacks the actual measured values in the study area, which affects the accuracy of the traceability results. Therefore, in the future research, the source apportionment of sulfate in the water environment should be studied by using multiple stable isotope techniques, such as, δD and δ^18^O of H_2_O and ^87^Sr/^86^Sr of Sr [84], combined with hydrochemical evolution theory and multivariate statistical techniques. At the same time, a source apportionment model should be established to quantitatively study the contribution of various sulfate sources to sulfate contamination in water environment, which can provide data support for scientific prevention and control of sulfate pollution in water environment.

## 7. Conclusions

The source apportionment of sulfate contamination in water environments has gone through the process of relying on the hydrogeochemical theory to the application of stable isotope techniques. In this paper, the application of stable isotope techniques to trace the sources of sulfate pollution at home and abroad is reviewed. In this paper, we have summarized the pretreatment methods of sulfur and oxygen isotopes in sulfate, which mainly include graphite reduction, fluorination, high temperature pyrolysis, chemical precipitation, triacid method, and flame heating method. The ranges of sulfur and oxygen isotopes values from potential sulfates sources were calculated. Furthermore, we have reviewed the research advances in the application of stable isotopes to identify sulfates sources in the water environment, which was developed from the qualitative method to quantitative method, and from single isotope to multiple isotope method. Due to the limitations of early technical conditions, only the δ^34^S isotope was used to identify the sulfate sources in water environment, and then the δ^34^S and δ^18^O double isotopes were developed to trace the sources. In recent years, the traceability researches have gradually evolved from qualitative to quantitative, and the accuracy has been improved gradually. However, due to the complexity of sulfate pollution sources and the isotope fractionation, using sulfur and oxygen isotopes alone cannot accurately identify the sources of sulfate pollution in water environment. Therefore, in order to provide a guarantee for the accurate identification of sulfate sources in the water environment the multi-isotopic traceability technology, combined with hydrochemistry and multivariate statistical analysis methods, and a source apportionment model are developed.
